# Associations of Education Level With Survival Outcomes and Treatment Receipt in Patients With Gastric Adenocarcinoma

**DOI:** 10.3389/fpubh.2022.868416

**Published:** 2022-06-09

**Authors:** Jiaxuan Xu, Shuhui Du, Xiaoqing Dong

**Affiliations:** Department of Hematology, The Affiliated Drum Tower Hospital of Nanjing University Medical School, Institute of Education, Nanjing University, Nanjing, China

**Keywords:** education level, gastric adenocarcinoma, prognosis, treatment receipt, SEER

## Abstract

**Background:**

It remains largely unclear how education level, an important socioeconomic factor, affects prognoses for patients with gastric adenocarcinoma (GAC). We aimed to demonstrate the associations between education level and clinical outcomes in patients with GAC.

**Methods:**

We included a total of 30,409 patients diagnosed with GAC from the Surveillance, Epidemiology, and End Results 18 registry database. Education level, household income, unemployment rate, poverty rate, insurance status, and marital status were selected as sociodemographic variables for the comprehensive analysis. Cox and logistic regression models, Kaplan–Meier curves, and subgroup analyses were the primary statistical methods employed.

**Results:**

A low level of education was correlated with less income, higher unemployment rates, and higher poverty rates (all *p* < 0.001). The multivariate Cox analysis indicated that a high education level was significantly associated with superior overall survival rates and cancer-specific survival rates in patients with GAC (both *p* < 0.001). We also corroborated favorable survival outcomes by high education level within almost every clinical and demographic subgroup. Furthermore, chemotherapy combined with surgery could markedly prolong the survival for all patients, including patients of stage IV cancer (both *p* < 0.001). By using multivariable logistic models, patients in counties with high education levels had a higher probability of chemotherapy receipt (*p* < 0.001). Contrarily, those in the counties with low levels of education were less likely to receive chemotherapy or undergo surgery (*p* < 0.001).

**Conclusions:**

Education level was identified and confirmed as an independent predictor of treatment and survival for GAC patients. Efforts are needed to provide effective interventions for those whose educational status is adverse.

## Introduction

Gastric cancer (GC) is one of the most frequent cause of tumors in the digestive system, with an estimated 26,560 new cases per year in the United States. It also remains the leading cause of cancer-related deaths (1). Gastric adenocarcinoma (GAC) is the most common type of gastric malignancy, accounting for ~95% of all types of GC (2). With advancements in therapeutic modalities, an improvement in GC patients' survival rate has been observed, whereas the 5-year overall survival (OS) rate is generally below 30% and the median relative survival rate is just 16 months (3). Moreover, prognoses for patients are highly dependent on the stage at diagnosis. The 5-year OS for those with distant metastases is <5% (3), and chemotherapy remains the preferred choice of treatment for the patients with an advanced GC (4). As a multifactorial disease, the environmental, demographic, and the genetic factors play pivotal roles in the etiology and lead to survival disparities in patients with GC (5). Given the very poor prognosis for patients with GC, the discovery of any factor that predicts better survival outcomes could be highly beneficial.

Socioeconomic status (SES) factors, including insurance status, marital status, income level, and education level, have been reported to influence the morbidity risk, treatment approaches, and long-term prognoses for patients with GC (6–15). Increased survival rates for patients with GC have been observed over the past few decades with a widening SES gap (11). Lower SES is linked to inferior survival rates (7, 11). In addition, patients whose SES is low have a lower probability of curative treatment allocations for gastrointestinal cancers, resulting in dismal prognoses (14). Besides, an adverse marital status (divorced or widowed), living alone, low education level, and low income increase the risks of all GC subtypes (8). A case–control study suggests that the education level could be a reliable and ideal single indicator to measure GC risk among several SES variables (15). Nevertheless, the impact of education level on the GC survival rate remains poorly understood.

Educational attainment is recognized as a crucial social determinant of diseases; it influences health through mechanisms such as biological aging, cognitive ability, and health behaviors (16). Education level has been demonstrated to impact treatment and prognoses in anal cancer, sinonasal cancer, and multiple myeloma (17–19). A nationwide cohort study in Sweden indicated that a high education level was associated with a greater likelihood of improved survival rates and curative treatment in 4,112 patients diagnosed with gastroesophageal cancer (10). Another investigation of 4,709 patients with stomach cancer in Sweden did not show a significant influence of education level on cancer survival rates (20). Considering the lack of such studies in the United States, as well as differences in education systems and patient characteristics between the two countries, we utilized the Surveillance, Epidemiology, and End Results (SEER) database to conduct a large-scale retrospective study in the United States. We investigated the effects of education level and other socioeconomic factors (e.g., income level, unemployment rate, insurance status, marital status) on the treatment receipt and clinical outcomes of patients with GAC.

## Materials and Methods

### Study Population

**Figure 1:**
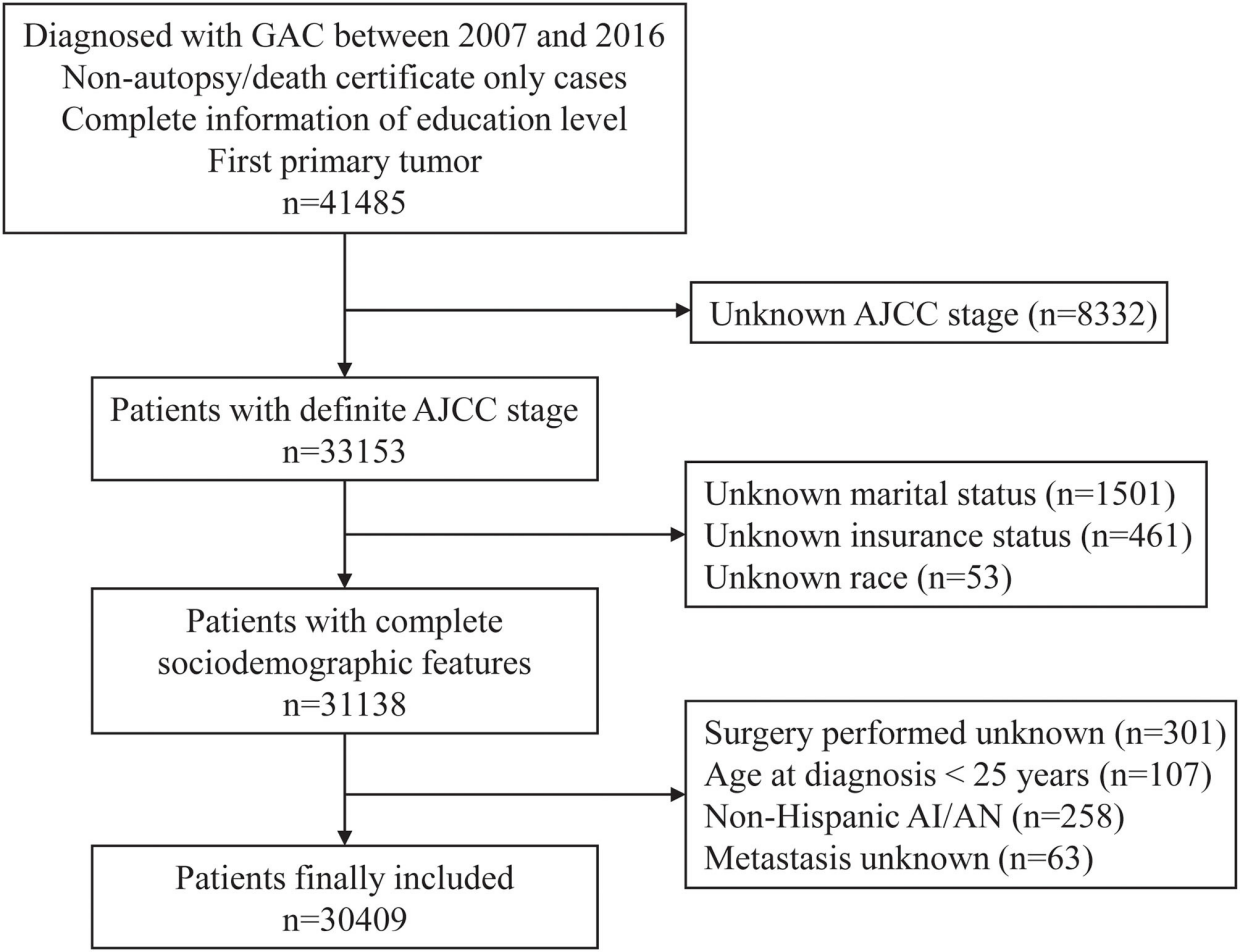
The flowchart for screening patients in the SEER database. GAC, gastric adenocarcinoma; AJCC, American Joint Committee on Cancer; AI/AN, American Indian/Alaska Native.

In this retrospective study, the patient data were extracted from the SEER database (18 cancer registries) *via* SEER^*^Stat software (version 8.3.9). Patients diagnosed with GAC were defined according to the International Classification of Disease for Oncology, Third Edition (ICD-O-3), histologic codes 8140, 8144, 8145, 8255, 8260, 8480, 8481, and 8490. The ICD-O-3 primary site code was C16 for stomach cancer. The flowchart for screening patients is presented in Figure 1. The patients were included preliminarily according to the following criteria: (a) Diagnosed with GAC from 1 January, 2007 to 31 December, 2016; (b) non-autopsy or death certificate only cases; (c) complete information regarding education level; and (d) first primary tumor. The following ineligible cases were excluded: (a) unclear American Joint Committee on Cancer (AJCC) stage; (b) unknown insurance status, marital status, or race; (c) unknown surgery information or metastasis status; (d) non-Hispanic American Indian/Alaska Native (NHAI/AN); and (e) age, <25 years, at diagnosis. Finally, a total of 30,409 patients were enrolled in the study cohort.

### Variable Selection

We selected the following sociodemographic and clinicopathological variables from the cohort: Age at diagnosis, sex, race, insurance status, marital status, SEER stage, AJCC stage, tumor grade, metastasis status, treatment approaches, median household income, unemployment rate, poverty rate, and education level. Race was categorized as follows: Non-Hispanic White (NHW), non-Hispanic Black (NHB), non-Hispanic Asian or Pacific Islander (NHAPI), and Hispanic. Marital status was grouped into four categories: Married, divorced, widowed, and single (never married, unmarried, or domestic partner). Insurance status was classified as insured, uninsured, or Medicaid. The SEER database contains county-level rather than patient-level socioeconomic attributes. The county-level household income, unemployment rate, and poverty rate were stratified into the following four quartiles: Quartile 1 (Q1, lowest), quartile 2 (Q2, lower), quartile 3 (Q3, higher), and quartile 4 (Q4, highest). The county-level educational attainment was measured by the percentage of those earning at least a bachelor's degree in the county where a patient was registered and divided into three groups: Q1 (low level, 25th percentile or lower, ≤23.96%), Q2 (moderate level, 25th−75th percentile, 23.96–39.07%), and Q3 (high level, 75th percentile or higher, ≥39.07%). Regarding the primary outcomes, OS was calculated as the interval from diagnosis to death from any cause. Cancer-specific survival (CSS) was calculated as the interval between diagnosis and death from GAC.

### Statistical Analyses

All analyses were performed using R 4.0.3 software. The distribution differences in baseline characteristics were compared with the Chi-squared test. Univariate and multivariate Cox regression analyses were conducted to determine the impacts of prognostic factors on survival outcomes. The univariate and multivariate logistic regression models were built to assess the effects of education level on treatment receipt. The hazard ratio (HR), odds ratio (OR), and 95% confidence interval (CI) were estimated for these results. The survival analysis was performed using Kaplan–Meier curves and evaluated by the log–rank test. In the subgroup analyses, forest plots described the influence of a high education level on prognosis compared with the influence of a low education level. A two-tailed *p* < 0.05 was considered statistically significant.

## Results

### Sociodemographic and Clinical Characteristics of Patients

**Figure 2:**
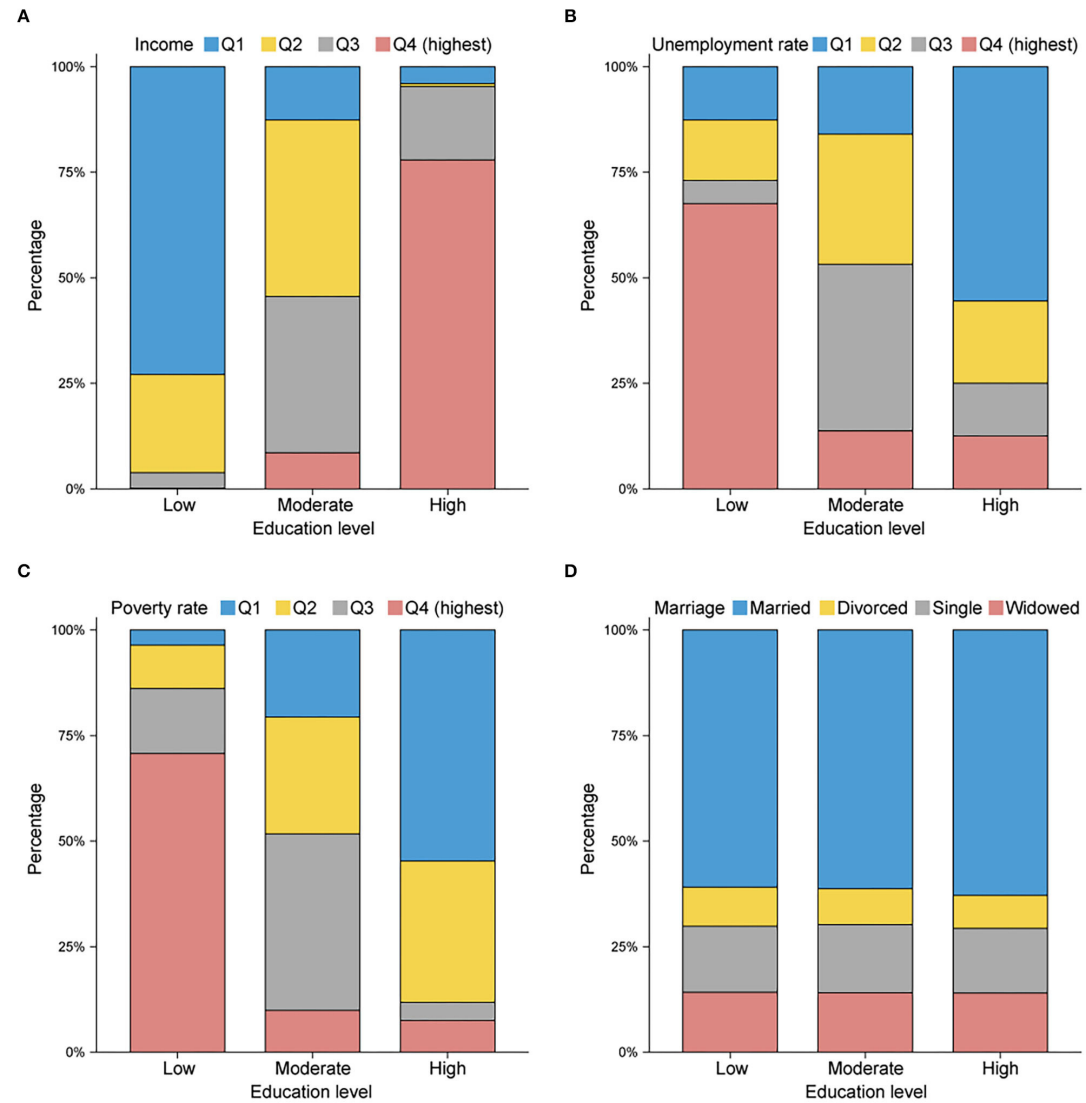
Histogram plots reflecting the proportions in **(A)** median household income, **(B)** unemployment rate, **(C)** poverty rate, and **(D)** marital status, stratified by county education level. Q1, quartile 1 (lowest); Q2, quartile 2 (lower); Q3, quartile 3 (higher); Q4, quartile 4 (highest). Counties with higher education levels tend to have higher average income levels and lower unemployment and poverty rates.

The baseline characteristics of 30,409 patients are summarized in Table 1. The patients were separated into the following three groups according to their education level: 7,626 in the low-level group, 14,823 in the moderate-level group, and 7,960 in the high-level group. The male patients represented a greater proportion than the female patients across the cohort (64.34% vs. 35.66%), and the proportion of males in the group with a low education level (66.39%) was higher than the proportion of males in the other two groups (*p* < 0.001). The percentage of those designated as NHAPI (23.74%) was conspicuously highest among those in the group with a high education level. Additionally, insured status was significantly larger for the patients in counties with high education levels (79.87%, *p* < 0.001), as were cases involving no metastasis (40.69%, *p* < 0.037). The patients in counties with high education levels were more likely to receive chemotherapy (57.83%, *p* < 0.001) and undergo surgery (49.26%, *p* < 0.001). As shown in Figure 2, counties with high education levels were associated with greater household income levels (Q4: 77.90%, *p* < 0.001), lower unemployment rates (Q1: 61.80%, *p* < 0.001), and lower poverty rates (Q1: 54.71%, *p* < 0.001), but the differences in marital status were not relatively obvious.

**Table 1:** Baseline characteristics of patients with GAC grouped by education level.

**Characteristics**	**All**	**Q1 (Low)**	**Q2 (Moderate)**	**Q3 (High)**	***p***
	***n* = 30,409**	***n* = 7,626**	***n* = 14,823**	***n* = 7,960**	
Age (years)					0.071
< =65	14,367 (47.25%)	3,670 (48.12%)	7,012 (47.30%)	3,685 (46.29%)	
>65	16,042 (52.75%)	3,956 (51.88%)	7,811 (52.70%)	4,275 (53.71%)	
Sex					<0.001
Male	19,566 (64.34%)	5,063 (66.39%)	9,465 (63.85%)	5,038 (63.29%)	
Female	10,843 (35.66%)	2,563 (33.61%)	5,358 (36.15%)	2,922 (36.71%)	
Race					<0.001
NHW	15,564 (51.18%)	4,385 (57.50%)	7,315 (49.35%)	3,864 (48.54%)	
NHB	3,877 (12.75%)	1,408 (18.46%)	1,626 (10.97%)	843 (10.59%)	
NHAPI	4,801 (15.79%)	349 (4.58%)	2,562 (17.28%)	1,890 (23.74%)	
Hispanic	6,167 (20.28%)	1,484 (19.46%)	3,320 (22.40%)	1,363 (17.12%)	
Insurance status					<0.001
Insured	23,648 (77.77%)	5,945 (77.96%)	11,345 (76.54%)	6,358 (79.87%)	
Uninsured	1,375 (4.52%)	321 (4.21%)	726 (4.90%)	328 (4.12%)	
Medicaid	5,386 (17.71%)	1,360 (17.83%)	2,752 (18.57%)	1,274 (16.01%)	
Marital status					0.033
Married	18,723 (61.57%)	4,645 (60.91%)	9,078 (61.24%)	5,000 (62.81%)	
Divorced	2,592 (8.52%)	703 (9.22%)	1,264 (8.53%)	625 (7.85%)	
Single	4,807 (15.81%)	1,194 (15.66%)	2,392 (16.14%)	1,221 (15.34%)	
Widowed	4,287 (14.10%)	1,084 (14.21%)	2,089 (14.09%)	1,114 (13.99%)	
SEER stage					0.008
Distant	13,381 (44.00%)	3,423 (44.89%)	6,544 (44.15%)	3,414 (42.89%)	
Localized	6,862 (22.57%)	1,765 (23.14%)	3,313 (22.35%)	1,784 (22.41%)	
Regional	10,166 (33.43%)	2,438 (31.97%)	4,966 (33.50%)	2,762 (34.70%)	
AJCC stage					0.134
I	8,152 (26.81%)	2,075 (27.21%)	3,920 (26.45%)	2,157 (27.10%)	
II	4,100 (13.48%)	979 (12.84%)	2,010 (13.56%)	1,111 (13.96%)	
III	3,823 (12.57%)	919 (12.05%)	1,891 (12.76%)	1,013 (12.73%)	
IV	14,334 (47.14%)	3,653 (47.90%)	7,002 (47.24%)	3,679 (46.22%)	
Tumor grade					<0.001
I	1,093 (3.59%)	285 (3.74%)	506 (3.41%)	302 (3.79%)	
II	6,910 (22.72%)	1,866 (24.47%)	3,294 (22.22%)	1,750 (21.98%)	
III	17,555 (57.73%)	4,150 (54.42%)	8,803 (59.39%)	4,602 (57.81%)	
IV	488 (1.60%)	132 (1.73%)	236 (1.59%)	120 (1.51%)	
Unknown	4,363 (14.35%)	1,193 (15.64%)	1,984 (13.38%)	1,186 (14.90%)	
Metastasis					0.037
No	17,681 (58.14%)	4,376 (57.38%)	8,584 (57.91%)	4,721 (59.31%)	
Yes	12,728 (41.86%)	3,250 (42.62%)	6,239 (42.09%)	3,239 (40.69%)	
Income					<0.001
Q1 (lowest)	7,749 (25.48%)	5,561 (72.92%)	1,871 (12.62%)	317 (3.98%)	
Q2	8,028 (26.40%)	1,774 (23.26%)	6,197 (41.81%)	57 (0.72%)	
Q3	7,148 (23.51%)	276 (3.62%)	5,487 (37.02%)	1,385 (17.40%)	
Q4 (highest)	7,484 (24.61%)	15 (0.20%)	1,268 (8.55%)	6,201 (77.90%)	
Unemployment rate					<0.001
Q1 (lowest)	8,249 (27.13%)	963 (12.63%)	2,367 (15.97%)	4,919 (61.80%)	
Q2	7,391 (24.31%)	1,093 (14.33%)	4,571 (30.84%)	1,727 (21.70%)	
Q3	7,369 (24.23%)	416 (5.46%)	5,846 (39.44%)	1,107 (13.91%)	
Q4 (highest)	7,400 (24.33%)	5,154 (67.58%)	2,039 (13.76%)	207 (2.60%)	
Poverty rate					<0.001
Q1 (lowest)	7,686 (25.28%)	275 (3.61%)	3,056 (20.62%)	4,355 (54.71%)	
Q2	7,548 (24.82%)	782 (10.25%)	4,102 (27.67%)	2,664 (33.47%)	
Q3	7,714 (25.37%)	1,175 (15.41%)	6,199 (41.82%)	340 (4.27%)	
Q4 (highest)	7,461 (24.54%)	5,394 (70.73%)	1,466 (9.89%)	601 (7.55%)	
Chemotherapy					<0.001
Yes	16,667 (54.81%)	4,084 (53.55%)	7,980 (53.84%)	4,603 (57.83%)	
No/Unknown	13,742 (45.19%)	3,542 (46.45%)	6,843 (46.16%)	3,357 (42.17%)	
Radiation					<0.001
Yes	8,529 (28.05%)	2,333 (30.59%)	3,946 (26.62%)	2,250 (28.27%)	
No	21,880 (71.95%)	5,293 (69.41%)	10,877 (73.38%)	5,710 (71.73%)	
Surgery					<0.001
Yes	14,699 (48.34%)	3,440 (45.11%)	7,338 (49.50%)	3,921 (49.26%)	
No	15,710 (51.66%)	4,186 (54.89%)	7,485 (50.50%)	4,039 (50.74%)	

*NHW, non-Hispanic White; NHB, non-Hispanic Black; NHAPI, non-Hispanic Asian or Pacific Islander; AJCC, American Joint Committee on Cancer*.

*Q1, low education level; Q2, moderate education level; Q3; high education level. p-Value is for comparisons among the three groups: Q1, Q2, and Q3*.

### Evaluation of Prognostic Indicators

**Figure 3:**
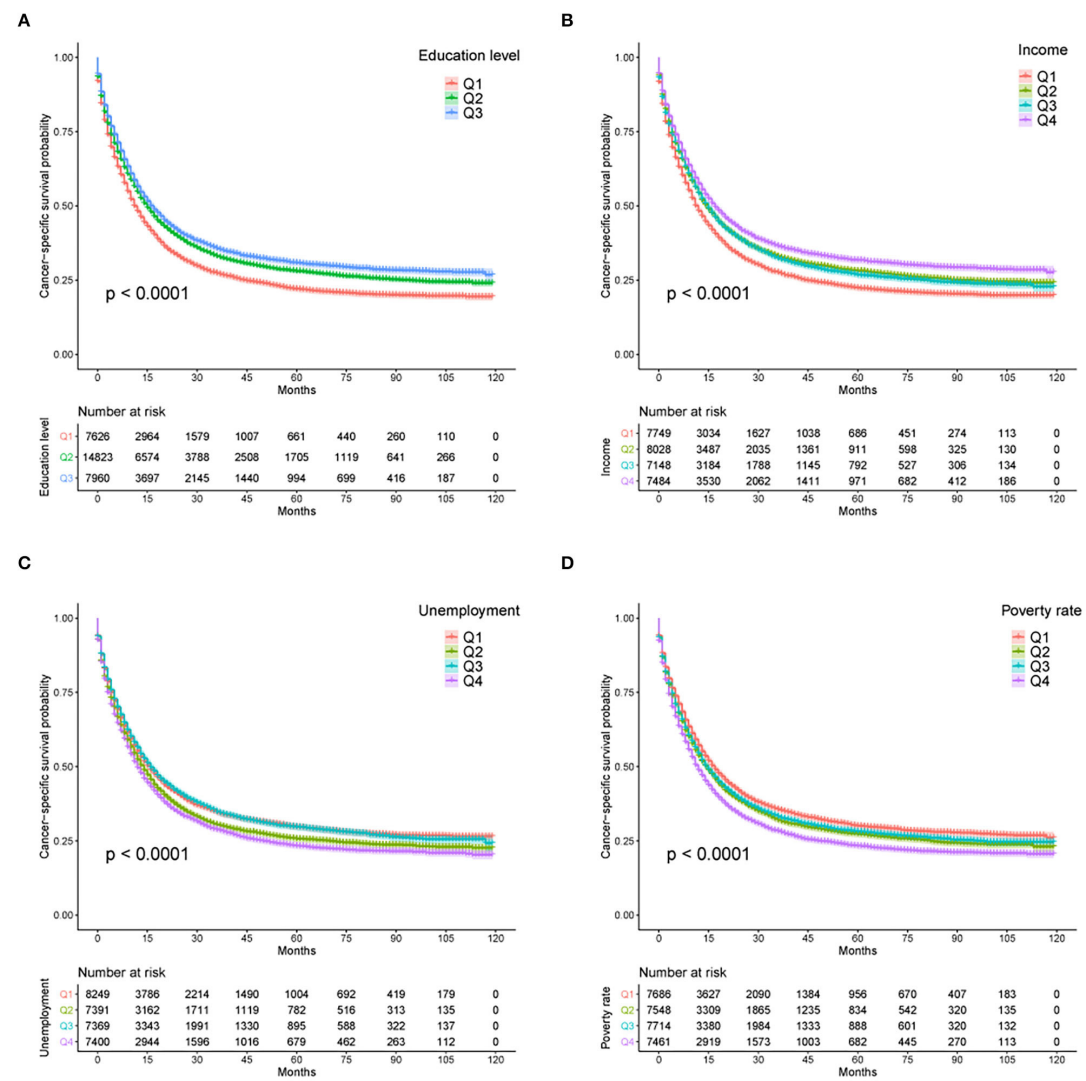
Kaplan–Meier survival curves for CSS according to **(A)** education level, **(B)** income, **(C)** unemployment rate, and **(D)** poverty rate. CSS, cancer-specific survival; U/M, uninsured/Medicaid; NHW, non-Hispanic White; NHB, non-Hispanic Black; NHAPI, non-Hispanic Asian or Pacific Islander.

From Kaplan–Meier survival curves stratified by the sociodemographic predictors, education level, income, unemployment, and poverty rate were linked to survival disparities in CSS (all *p* < 0.001, Figure 3) and OS (all *p* < 0.001, Supplementary Figure 1). Then, a univariate Cox analysis identified the prognostic value of each factor in the entire cohort (Table 2). All variables except sex variable proved to be significant in predicting OS and CSS and were further included in the multivariable model (Table 3). The multivariate Cox regression analysis confirmed education level as an independent predictor in the survival of patients with GAC. A high education level was significantly associated with superior OS (HR: 0.915, *p* = 0.005) and CSS (HR: 0.907, *p* = 0.004) when compared with a low education level. A moderate education level was also correlated with longer OS (HR: 0.926, *p* < 0.001) and CSS (HR: 0.915, *p* < 0.001) when compared with a low level of education. Compared with the lowest income level, the highest income level was related to better outcomes in both OS (HR: 0.902, *p* = 0.016) and CSS (HR: 0.909, *p* = 0.037). With regard to other sociodemographic factors, age more than 65 years, NHAPI or Hispanic designation, Medicaid status, and unmarried status were independent prognostic indicators of clinical survival. In addition, less mortality risks were observed in patients with localized stage, AJCC stage I, and Grade I tumors (all *p* < 0.001). The patients who did not receive chemotherapy or surgery experienced worse OS and CSS rates (all *p* < 0.001), and radiotherapy showed no significant effect on survival.

**Table 2:** Univariate cox analysis of OS and CSS.

**Characteristics**	**Levels**	**OS**			**CSS**		
		**HR**	**95% CI**	***p***	**HR**	**95% CI**	***p***
Age	≤65 years	Ref			Ref		
	>65 years	1.207	1.176–1.239	<0.001	1.103	1.072–1.134	<0.001
Sex	Male	Ref			Ref		
	Female	1.026	0.999–1.054	0.064	1.028	0.998–1.058	0.067
Race	NHW	Ref			Ref		
	NHB	1.052	1.011–1.094	0.013	1.023	0.980–1.068	0.301
	NHAPI	0.701	0.674–0.730	<0.001	0.684	0.656–0.714	<0.001
	Hispanic	0.972	0.939–1.006	0.103	0.965	0.930–1.001	0.055
Insurance	Insured	Ref			Ref		
	Uninsured	1.276	1.196–1.361	<0.001	1.303	1.217–1.394	<0.001
	Medicaid	1.127	1.089–1.166	<0.001	1.095	1.055–1.136	<0.001
Marriage	Married	Ref			Ref		
	Divorced	1.172	1.118–1.228	<0.001	1.144	1.088–1.203	<0.001
	Single	1.238	1.193–1.284	<0.001	1.215	1.169–1.264	<0.001
	Widowed	1.431	1.379–1.486	<0.001	1.337	1.284–1.392	<0.001
SEER stage	Distant	Ref			Ref		
	Localized	0.198	0.190–0.206	<0.001	0.150	0.143–0.157	<0.001
	Regional	0.329	0.319–0.339	<0.001	0.307	0.298–0.317	<0.001
AJCC stage	I	Ref			Ref		
	II	1.298	1.235–1.364	<0.001	1.520	1.437–1.608	<0.001
	III	1.852	1.765–1.943	<0.001	2.258	2.139–2.382	<0.001
	IV	4.602	1.765–1.943	<0.001	5.866	5.627–6.116	<0.001
Tumor grade	I	Ref			Ref		
	II	1.566	1.435–1.710	<0.001	1.753	1.584–1.941	<0.001
	III	2.101	1.931–2.287	<0.001	2.514	2.278–2.774	<0.001
	IV	1.843	1.612–2.107	<0.001	2.231	1.926–2.585	<0.001
	Unknown	2.869	2.624–3.137	<0.001	3.410	3.076–3.779	<0.001
Metastasis	No	Ref			Ref		
	Yes	3.793	3.689–3.899	<0.001	4.281	4.156–4.410	<0.001
Chemotherapy	Yes	Ref			Ref		
	No/Unknown	1.291	1.258–1.326	<0.001	1.199	1.166–1.234	<0.001
Radiation	Yes	Ref			Ref		
	No	1.442	1.400–1.486	<0.001	1.425	1.381–1.471	<0.001
Surgery	Yes	Ref			Ref		
	No	4.174	4.056–4.295	<0.001	4.562	4.422–4.705	<0.001
Income	Q1 (lowest)	Ref			Ref		
	Q2	0.842	0.812–0.872	<0.001	0.849	0.817–0.882	<0.001
	Q3	0.854	0.824–0.886	<0.001	0.861	0.828–0.895	<0.001
	Q4 (highest)	0.762	0.734–0.791	<0.001	0.765	0.736–0.796	<0.001
Unemployment	Q1 (lowest)	Ref			Ref		
	Q2	1.115	1.075–1.156	<0.001	1.110	1.068–1.154	<0.001
	Q3	1.002	0.966–1.040	0.903	0.990	0.951–1.030	0.606
	Q4 (highest)	1.198	1.156–1.243	<0.001	1.184	1.139–1.231	<0.001
Poverty rate	Q1 (lowest)	Ref			Ref		
	Q2	1.103	1.062–1.144	<0.001	1.100	1.057–1.144	<0.001
	Q3	1.070	1.031–1.111	<0.001	1.076	1.034–1.119	<0.001
	Q4 (highest)	1.262	1.216–1.309	<0.001	1.245	1.197–1.295	<0.001
Education level	Q1 (low)	Ref			Ref		
	Q2 (moderate)	0.843	0.816–0.869	<0.001	0.842	0.815–0.871	<0.001
	Q3 (high)	0.776	0.749–0.805	<0.001	0.778	0.748–0.809	<0.001

*HR, hazard ratio; CI, confidence interval; NHW, non-Hispanic White; NHB, non-Hispanic Black; NHAPI, non-Hispanic Asian or Pacific Islander; AJCC, American Joint Committee on Cancer*.

**Table 3:** Multivariate cox analysis of OS and CSS.

**Characteristics**	**Levels**	**OS**			**CSS**		
		**HR**	**95% CI**	***p***	**HR**	**95% CI**	***p***
Age	≤65 years	Ref			Ref		
	>65 years	1.275	1.238–1.313	<0.001	1.192	1.156–1.230	<0.001
Race	NHW	Ref			Ref		
	NHB	0.982	0.942–1.024	0.397	0.960	0.917–1.004	0.072
	NHAPI	0.819	0.785–0.855	<0.001	0.813	0.776–0.851	<0.001
	Hispanic	0.940	0.906–0.976	0.001	0.924	0.888–0.961	<0.001
Insurance	Insured	Ref			Ref		
	Uninsured	1.063	0.994–1.136	0.075	1.043	0.972–1.119	0.242
	Medicaid	1.062	1.024–1.101	0.001	1.027	0.988–1.068	0.181
Marriage	Married	Ref			Ref		
	Divorced	1.111	1.059–1.165	<0.001	1.082	1.029–1.139	0.002
	Single	1.113	1.071–1.156	<0.001	1.086	1.043–1.131	<0.001
	Widowed	1.180	1.135–1.228	<0.001	1.155	1.107–1.205	<0.001
SEER stage	Distant	Ref			Ref		
	Localized	0.621	0.550–0.702	<0.001	0.531	0.465–0.606	<0.001
	Regional	1.057	0.962–1.161	0.252	1.035	0.938–1.143	0.493
AJCC stage	I	Ref			Ref		
	II	1.257	1.159–1.364	<0.001	1.305	1.192–1.429	<0.001
	III	1.866	1.722–2.023	<0.001	2.006	1.835–2.193	<0.001
	IV	2.456	2.243–2.690	<0.001	2.670	2.418–2.949	<0.001
Tumor grade	I	Ref			Ref		
	II	1.240	1.136–1.355	<0.001	1.307	1.180–1.448	<0.001
	III	1.644	1.509–1.792	<0.001	1.806	1.635–1.995	<0.001
	IV	1.692	1.479–1.936	<0.001	1.914	1.651–2.219	<0.001
	Unknown	1.450	1.325–1.587	<0.001	1.571	1.415–1.743	<0.001
Metastasis	No	Ref			Ref		
	Yes	1.165	1.056–1.285	0.002	1.155	1.043–1.280	0.006
Chemotherapy	Yes	Ref			Ref		
	No/Unknown	2.373	2.300–2.448	<0.001	2.379	2.301–2.458	<0.001
Radiation	Yes	Ref			Ref		
	No	0.997	0.965–1.030	0.859	0.989	0.955–1.024	0.529
Surgery	Yes	Ref			Ref		
	No	3.112	3.000–3.228	<0.001	3.235	3.110–3.366	<0.001
Income	Q1 (lowest)	Ref			Ref		
	Q2	0.944	0.896–0.993	0.027	0.946	0.895–0.999	0.047
	Q3	0.938	0.880–1.000	0.050	0.944	0.882–1.010	0.097
	Q4 (highest)	0.902	0.829–0.981	0.016	0.909	0.831–0.994	0.037
Unemployment	Q1 (lowest)	Ref			Ref		
	Q2	1.057	1.014–1.102	0.008	1.050	1.005–1.098	0.028
	Q3	0.953	0.907–1.001	0.057	0.935	0.887–0.986	0.013
	Q4 (highest)	0.996	0.946–1.049	0.887	0.979	0.927–1.034	0.453
Poverty rate	Q1 (lowest)	Ref			Ref		
	Q2	1.010	0.968–1.053	0.659	1.015	0.970–1.061	0.529
	Q3	0.993	0.928–1.063	0.840	1.025	0.954–1.102	0.502
	Q4 (highest)	1.057	0.984–1.135	0.126	1.064	0.986–1.148	0.108
Education level	Q1 (low)	Ref			Ref		
	Q2 (moderate)	0.926	0.885–0.969	0.001	0.915	0.872–0.960	<0.001
	Q3 (high)	0.915	0.860–0.973	0.005	0.907	0.849–0.969	0.004

*HR, hazard ratio; CI, confidence interval; NHW, non-Hispanic White; NHB, non-Hispanic Black; NHAPI, non-Hispanic Asian or Pacific Islander; AJCC, American Joint Committee on Cancer*.

### Subgroup Analysis for Education Level

**Figure 4:**
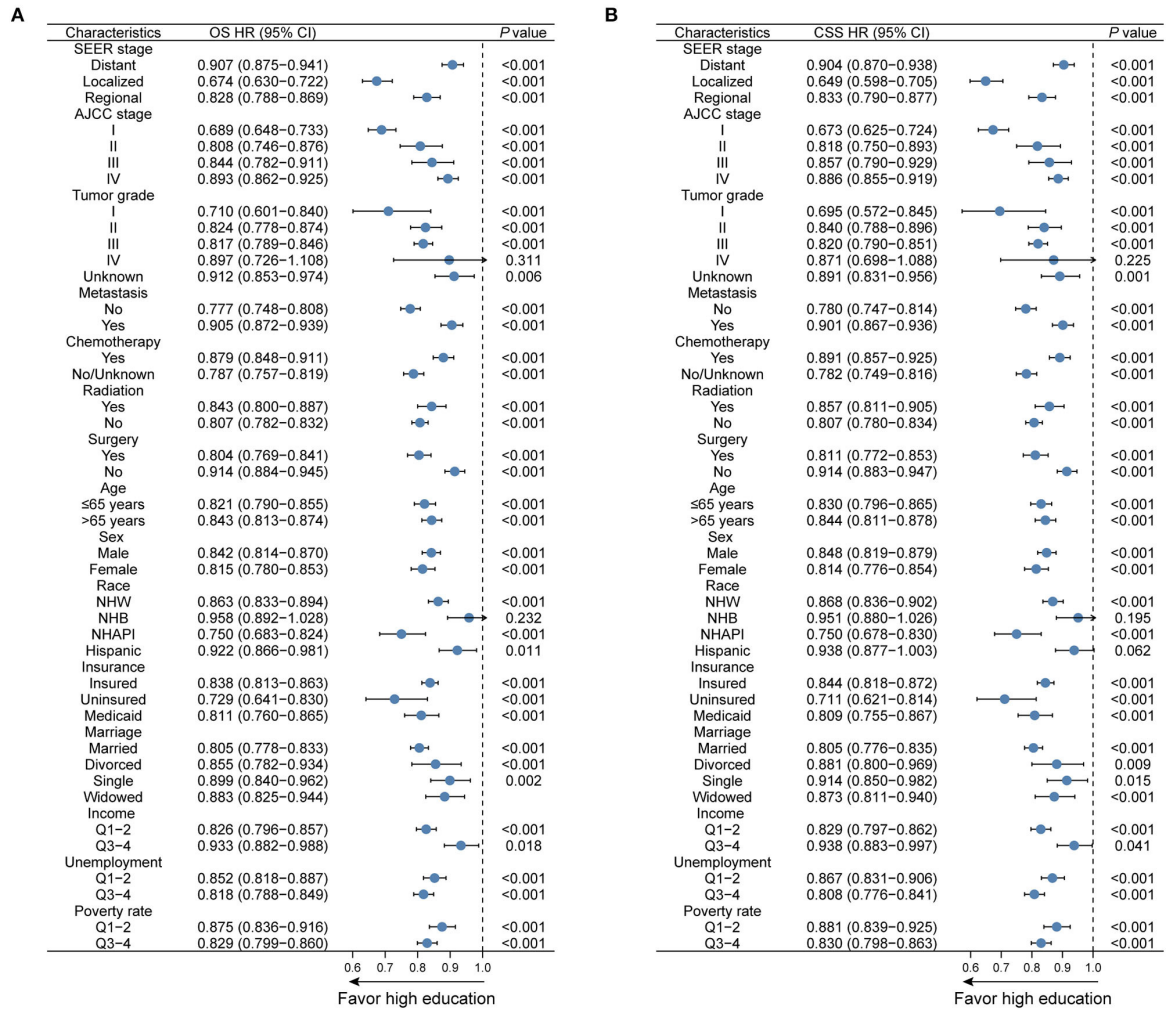
Subgroup analyses regarding education level for **(A)** OS and **(B)** CSS in the overall cohort. OS, overall survival; CSS, cancer-specific survival; HR, hazard ratio; CI, confidence interval; NHW, non-Hispanic White; NHB, non-Hispanic Black; NHAPI, non-Hispanic Asian or Pacific Islander.

To verify the prognostic impacts of education level on survival in different subgroups, Kaplan–Meier curves stratified by education level were exhibited. As expected, a low level of education was distinctly correlated with adverse OS outcomes in subgroups, including age more than 65 years, NHW, unmarried status, uninsured or Medicaid status, distant stage, AJCC stage IV, Grade II–IV tumors, and metastatic status (all *p* < 0.001, Supplementary Figure 2). The unfavorable effects of education level on CSS were also pronounced (all *p* < 0.001, Supplementary Figure 3). Moreover, we divided education level into a dichotomous variable by the median (31.23%) and further outlined the influence of education level for each subgroup. Forest plots revealed that a higher level of education markedly favored survival prognoses in the vast majority of subgroups (Figure 4). Nonetheless, the favorable effects of higher education levels were not appreciable in either OS or CSS for NHBs or patients with Grade IV tumors (both *p* > 0.05).

### Influence of Education Level on Treatment

**Figure 5:**
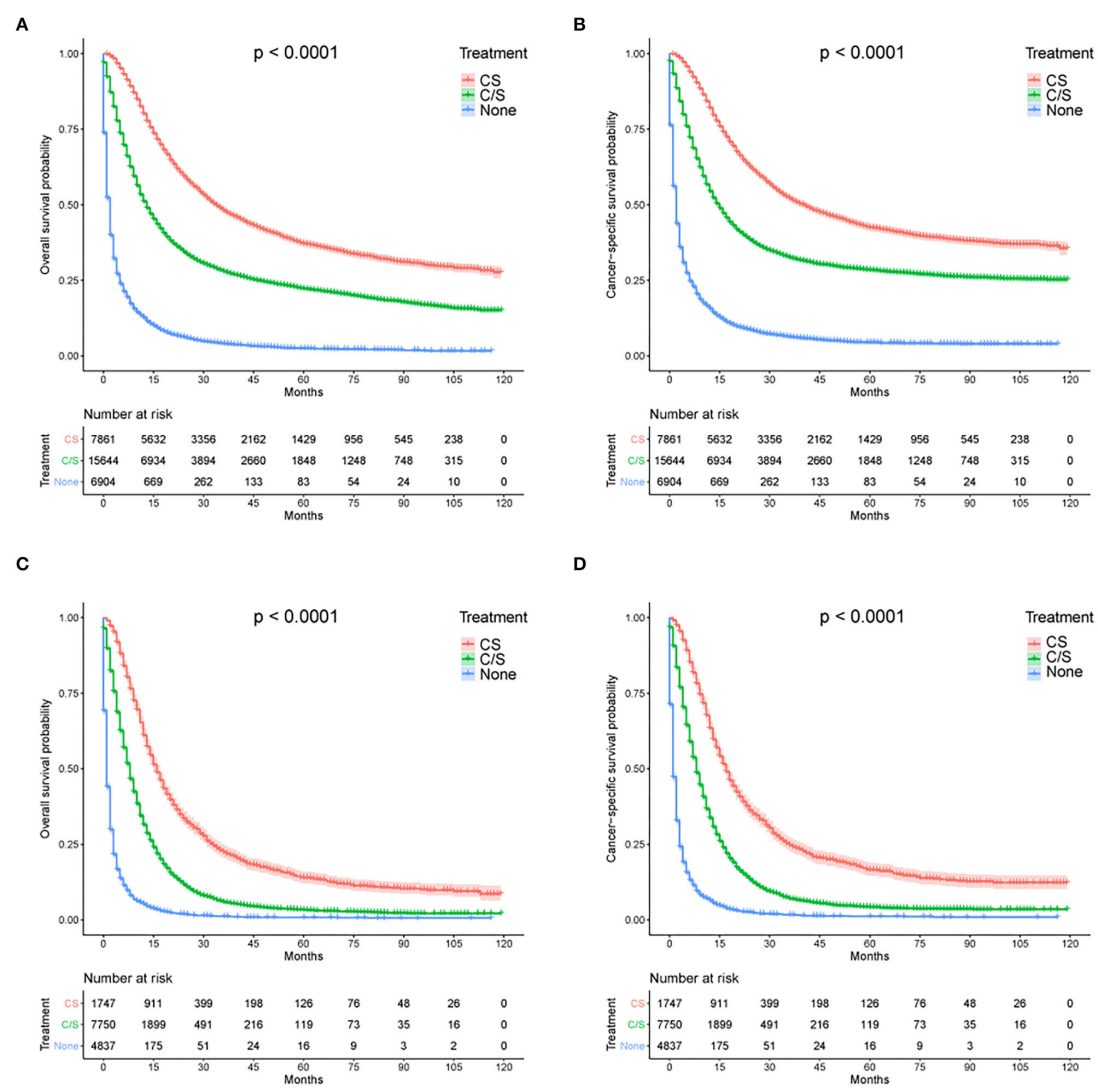
Survival analysis of treatment options for **(A)** OS and **(B)** CSS among all patients with GAC; Survival analysis of treatment options for **(C)** OS and **(D)** CSS among AJCC stage IV tumor patients. CS, chemotherapy combined with surgery; C/S, chemotherapy or surgery alone; None, no chemotherapy or surgery.

Given the dismal prognosis for GAC, we examined the therapeutic benefits of different treatment modalities, including chemotherapy plus surgery (CS), chemotherapy or surgery alone (C/S), and no chemotherapy or surgery (None). In all patients with GAC, the median survival times for these modalities were 34, 13, and 2 months, respectively, for OS (*p* < 0.001, Figure 5A) and 40, 15, and 2 months, respectively, for CSS (*p* < 0.001, Figure 5B). Considering that AJCC stage IV tumor confers the poorest survival outcomes, we also performed the same analysis for patients with AJCC stage IV tumor. The median survival months based on the CS, C/S, and “None” modalities were 16, 8, and 1, respectively, for OS (*p* < 0.001, Figure 5C) and 17, 8, and 1, respectively, for CSS (*p* < 0.001, Figure 5D). Subsequently, a logistic regression model was applied to determine the factors affecting treatment receipt by patients with AJCC stage IV tumor. Through a multivariate analysis (Table 4), a lower probability of receiving chemotherapy was correlated with the following indicators: Age, race, insurance status, marital status, metastatic status, poverty rate, and education level. Table 5 shows that age, insurance status, marital status, SEER stage, metastatic status, poverty rate, and education level could significantly influence the odds of receiving no treatment (no chemotherapy or surgery) in the multivariate model. Notably, a high level of education was independently associated with a higher receipt of chemotherapy (OR: 1.295, *p* = 0.002) and a lower probability of no treatment (OR: 0.814, *p* = 0.023). Even though radiotherapy was not significant in the multivariate Cox analysis, we described the differences in radiotherapy receipt by education level (Supplementary Table 1), given the role of radiotherapy in the standard of health care. Our results showed that no statistical significance was observed in the receipt of radiation among different education levels.

**Table 4:** Univariate and multivariate logistic regression analysis of chemotherapy receipt in AJCC stage IV patients.

**Characteristics**	**Levels**	**Crude OR**	**95% CI**	***p***	**Adjusted OR**	**95% CI**	***p***
Age	≤ 65 years	Ref			Ref		
	>65 years	0.380	0.355–0.407	<0.001	0.353	0.327–0.381	<0.001
Sex	Male	Ref			Ref		
	Female	0.831	0.776–0.890	<0.001	1.008	0.934–1.088	0.843
Race	NHW	Ref			Ref		
	Hispanic	0.941	0.866–1.024	0.157	0.922	0.837–1.015	0.097
	NHAPI	0.874	0.790–0.967	0.009	0.826	0.737–0.926	0.001
	NHB	0.769	0.694–0.851	<0.001	0.869	0.776–0.972	0.014
Insurance	Insured	Ref			Ref		
	Medicaid	0.731	0.672–0.796	<0.001	0.685	0.623–0.754	<0.001
	Uninsured	0.639	0.557–0.734	<0.001	0.439	0.378–0.509	<0.001
Marriage	Married	Ref			Ref		
	Divorced	0.775	0.687–0.874	<0.001	0.756	0.667–0.858	<0.001
	Single	0.658	0.602–0.720	<0.001	0.617	0.560–0.680	<0.001
	Widowed	0.299	0.269–0.333	<0.001	0.434	0.386–0.488	<0.001
SEER stage	Distant	Ref			Ref		
	Regional	1.258	1.121–1.411	<0.001	0.761	0.552–1.049	0.095
Tumor grade	I	Ref					
	II	0.913	0.692–1.206	0.523			
	III	1.045	0.798–1.369	0.749			
	IV	0.871	0.592–1.281	0.483			
	Unknown	0.819	0.621–1.081	0.159			
Metastasis	No	Ref			Ref		
	Yes	0.758	0.681–0.845	<0.001	0.542	0.400–0.733	<0.001
Income	Q1 (lowest)	Ref			Ref		
	Q2	1.057	0.965–1.158	0.233	1.064	0.927–1.221	0.379
	Q3	1.324	1.204–1.457	<0.001	1.056	0.889–1.256	0.534
	Q4 (highest)	1.355	1.232–1.490	<0.001	0.918	0.731–1.152	0.460
Unemployment	Q1 (lowest)	Ref			Ref		
	Q2	0.940	0.855–1.032	0.195	1.047	0.936–1.170	0.422
	Q3	0.807	0.735–0.886	<0.001	0.999	0.874–1.142	0.990
	Q4 (highest)	0.767	0.699–0.841	<0.001	0.972	0.845–1.118	0.691
Poverty rate	Q1 (lowest)	Ref			Ref		
	Q2	0.841	0.764–0.925	<0.001	0.834	0.744–0.935	0.002
	Q3	0.703	0.640–0.773	<0.001	0.731	0.609–0.879	0.001
	Q4 (highest)	0.698	0.635–0.768	<0.001	0.776	0.639–0.944	0.011
Education level	Q1 (low)	Ref			Ref		
	Q2 (moderate)	1.125	1.038–1.220	0.004	1.079	0.955–1.219	0.220
	Q3 (high)	1.373	1.251–1.508	<0.001	1.295	1.095–1.531	0.002

*OR, odd ratio; CI, confidence interval; NHW, non-Hispanic White; NHB, non-Hispanic Black; NHAPI, non-Hispanic Asian or Pacific Islander*.

**Table 5:** Univariate and multivariate logistic regression analysis of no treatment (no chemotherapy or surgery) in AJCC stage IV patients.

**Characteristics**	**Levels**	**Crude OR**	**95% CI**	***p***	**Adjusted OR**	**95% CI**	***p***
Age	≤65 years	Ref			Ref		
	>65 years	2.232	2.080–2.395	<0.001	2.556	2.356–2.774	<0.001
Sex	Male	Ref			Ref		
	Female	1.106	1.029–1.188	0.006	0.934	0.861–1.012	0.096
Race	NHW	Ref			Ref		
	Hispanic	0.972	0.890–1.061	0.522	1.045	0.944–1.157	0.396
	NHAPI	0.927	0.833–1.031	0.163	1.054	0.933–1.190	0.397
	NHB	1.223	1.101–1.359	<0.001	1.120	0.996–1.260	0.058
Insurance	Insured	Ref			Ref		
	Medicaid	1.239	1.135–1.354	<0.001	1.353	1.224–1.495	<0.001
	Uninsured	1.680	1.462–1.931	<0.001	2.329	1.997–2.716	<0.001
Marriage	Married	Ref			Ref		
	Divorced	1.338	1.180–1.516	<0.001	1.363	1.194–1.557	<0.001
	Single	1.606	1.465–1.762	<0.001	1.712	1.546–1.895	<0.001
	Widowed	2.779	2.504–3.084	<0.001	2.140	1.900–2.409	<0.001
SEER stage	Distant	Ref			Ref		
	Regional	0.134	0.109–0.164	<0.001	0.451	0.292–0.698	<0.001
Tumor grade	I	Ref			Ref		
	II	1.014	0.761–1.352	0.924	1.063	0.784–1.442	0.695
	III	0.833	0.630–1.102	0.200	1.026	0.763–1.381	0.863
	IV	0.742	0.491–1.123	0.158	0.949	0.611–1.473	0.815
	Unknown	1.476	1.109–1.964	0.008	1.607	1.187–2.177	0.002
Metastasis	No	Ref			Ref		
	Yes	6.605	5.505–7.925	<0.001	3.787	2.576–5.567	<0.001
Income	Q1 (lowest)	Ref			Ref		
	Q2	0.876	0.797–0.963	0.006	0.899	0.777–1.039	0.149
	Q3	0.804	0.728–0.887	<0.001	0.944	0.787–1.133	0.538
	Q4 (highest)	0.754	0.683–0.833	<0.001	1.022	0.805–1.299	0.857
Unemployment	Q1 (lowest)	Ref			Ref		
	Q2	1.014	0.919–1.119	0.780	0.888	0.789–0.998	0.047
	Q3	1.085	0.985–1.196	0.099	0.918	0.798–1.057	0.235
	Q4 (highest)	1.235	1.121–1.359	<0.001	1.023	0.884–1.185	0.759
Poverty rate	Q1 (lowest)	Ref			Ref		
	Q2	1.127	1.020–1.245	0.019	1.140	1.011–1.285	0.032
	Q3	1.219	1.105–1.345	<0.001	1.258	1.037–1.526	0.020
	Q4 (highest)	1.308	1.184–1.444	<0.001	1.155	0.941–1.419	0.168
Education level	Q1 (low)	Ref			Ref		
	Q2 (moderate)	0.885	0.814–0.962	0.004	0.961	0.846–1.092	0.543
	Q3 (high)	0.762	0.692–0.840	<0.001	0.814	0.682–0.972	0.023

*OR, odd ratio; CI, confidence interval; NHW, non-Hispanic White; NHB, non-Hispanic Black; NHAPI, non-Hispanic Asian or Pacific Islander*.

## Discussion

Of note, SES proved to play a vital role in the pathogenesis of the disease and survival rate of patients with GC (6, 7, 11), yet there remains a paucity of evidence on the prognostic effects resulting from education level. It is necessary to elaborate on this issue with a large population-based study. Through SEER data, we clarified that low education level was significantly linked to diminished survival rates in patients with GAC, serving as an independent and robust predictor. This view was strengthened by evidence from the subgroup analysis. Moreover, a higher risk for receiving no treatment was observed among patients in counties with low education levels, which explained their poor prognoses to some extent.

Our results confirm that a high level of education confers survival advantages, regardless of other variables. This finding is consistent with that of a previous study on gastroesophageal tumors (10). By contrast, our study included more recently diagnosed patients, larger sample sizes, and a longer follow-up period. The classifications for educational status were determined rationally by referring to a specialized index of educational degrees. Two earlier studies based on the European population also reported that a higher education level was a favorable prognostic factor in patients with GC (21, 22), which further supports our conclusion. However, these two studies only assessed the impact of simple SES on the survival of patients with GC; clinical factors were not considered. We included detailed clinical variables and confirmed the importance of education level after adjusting for covariates. Moreover, we conducted comprehensive subgroup analyses to fully describe that a low education level carries a higher risk for both OS and CSS in almost every subgroup, highlighting its broad applicability as a prognostic predictor. Nevertheless, we noted that the effect of education category was not so significant for NHB and patients with Grade IV tumor, most likely because of the small sample size of the NHB group. For the latter, a higher tumor grade was positively correlated with the development of a distant metastasis. leading to very poor survival rates for patients with GC (23). This finding could potentially attenuate the effects of education.

A prognosis is largely dependent on treatment modalities and types. As expected, in this study, patients undergoing a combination of surgical resection and chemotherapy experienced the best survival benefits; specifically, gastrectomy performed after chemotherapy led to superior outcomes (24, 25). The survival trends also persisted in stage IV patients, for whom prognoses were quite dismal (as illustrated by the Kaplan–Meier curves). For this population, palliative chemotherapy with supportive care remains the main treatment modality (4, 26). We performed a logistic regression analysis to validate the role of education in the use of cancer treatments. Here, we report for the first time that a low education level is strongly associated with a lack of therapy in patients with stage IV GC, and this finding complements the two other studies on non-advanced or regional GC (14, 27). However, radiotherapy was not associated with significant survival differences, which is likely due to the low sensitivity of GAC to radiotherapy. Currently, the role of radiotherapy in adjuvant treatment or palliative treatment for GC is less certain (28, 29).

Similar findings have also been reported for other tumor types (10, 17–19), indicating that education disparities are imperative public health concerns. Thus, we were interested in the underlying effects of individual education levels for patients with cancer. First, patients with high education levels tend to be in high SES categories, with easier access to health care and greater financial resources, which typically guarantee better health outcomes. Evidence also supports financial income as positively related to health status (30). Second, high educational attainment has positive impacts on vigorous physical exercise regimens and negative impacts on smoking, poor diet, sedentary habits, and other unhealthy behaviors (31). Convincing data show that appropriate diet, physical activities, and normal BMI have survival benefits for cancers (32), as they are associated with such biological hallmarks as immune responses, epigenetic regulation, and rhythm disorders (33). Third, highly educated patients have higher levels of health awareness (i.e., understanding medical information and receiving effective interventions) (34). Fourth, from a psychological perspective, the patients with higher education levels are less likely to become depressed or low-spirited (35). Depression or psychosocial stressors can drive tumor progression and cause worse outcomes through a large number of biobehavioral pathways (36). Fifth, employment rates and income levels are both higher among the better educated patients, which may facilitate their access to advanced treatment regimens with superior continuity and completion. However, the inadequacy is that the examined variable is not the real measure of individual education levels. Our explanations are just based on the putative correlation between county-level education and individual-level education. In county-level terms, counties characterized by high SES have greater medical resources and services compared to those characterized by low SES. Counties with sufficient resources also have greater financial support, more Medicaid funding and programs, and a higher prevalence of academic centers, clinical trials, and collaborations with pharmaceutical companies. Moreover, counties known for high SES have populations that are wealthier, more educated, and more likely to be employed and insured, and these differences in the composition of patient demographics may contribute to the positive effects on survival.

In addition to education level, we identified other demographic factors associated with the treatment and survival for the patients with GAC. Married patients had the lowest rate for no treatment and the highest acceptance rate of chemotherapy compared with the three other unmarried groups. A good marriage may provide adequate economic resources and social support as the basis for treatment regimens. On the contrary, widowed patients suffered the worst survival rates, possibly because they were less likely to receive treatment and had less social support, higher levels of loneliness, and an inferior insurance status. We also point out that uninsured or Medicaid patients had a significantly higher risk of no treatment. One reasonable explanation for this finding is that an uninsured or Medicaid status is more likely to be linked to poverty, low education levels, and other unfavorable SES factors. Interestingly, those with high income levels tended be less vulnerable to low education levels, according to the forest plots. This means that the adverse effect of a low level of education can possibly be compensated by increasing an individual's revenue. Notably, the beneficial effects of a high education level were the most obvious in the early stages of tumor development, including localized stage, AJCC stage I, and Grade I tumors, and non-metastatic status. The potential reasons for the favorable prognoses of highly educated individuals are advanced health consciousness, earlier diagnosis, and more effective interventions.

The present study has several limitations. First, sociodemographic variables provided by the SEER database were at the county-level rather than the individual level. The principal conclusions were dependent on an investigation at the community level; personal information was lacking. Nevertheless, this is still a reliable measure with practical implications for assessing SES, which has always been widely applied in SEER-based socioeconomic studies. Second, our results only reflect part of the affected population in the United States, and it remains uncertain whether these findings can be applied to other regions of the world. Lastly, we have provided comprehensive evidence of associations between education level and GC but no formal proof of causality. Further statistical methods need to be employed to evaluate casual effects.

While patient knowledge and education levels will remain relatively unchanged among older adults, the adverse sociodemographic status attributed to education levels cannot be ignored. More effective interventions are warranted to mitigate the unfavorable effects from low education levels and SES. Clinicians should enhance health education for patients to promote their health perceptions and behaviors. Governments and medical institutions are also expected to provide equal access to health care resources and services for those vulnerable groups. Income level, insurance status, marital status, and other socioeconomic elements are also involved in multifactor interactions of the disease treatment process. Long-term tracking in the dynamic changes of these factors will be informative for explaining the mechanisms.

To conclude, the education level is sufficiently established as an independent predictor for survival differences in patients with GAC. Our study indicates that the higher education levels may offer greater survival benefits and increase chemotherapy receipt. With predictions based on the education levels, designing personalized and suitable treatment plans in clinical practice is a promising approach.

## Data Availability Statement

The original contributions presented in the study are included in the article/Supplementary Material, further inquiries can be directed to the corresponding author/s.

## Author Contributions

All authors made a significant contribution to the work reported, whether in the conception, study design, execution, acquisition of data, analysis, and interpretation, or in all these areas. Additionally, all authors took part in drafting, revising, and reviewing the article, giving final approval to the version to be published, agreeing on the journal to which the article has been submitted, and remaining accountable for all aspects of the work.

## Funding

This work was supported by the grant from the Medical Science and Technology Development Foundation, Nanjing Department of Health (YKK20083).

## Conflict of Interest

The authors declare that the research was conducted in the absence of any commercial or financial relationships that could be construed as a potential conflict of interest.

## Publisher's Note

All claims expressed in this article are solely those of the authors and do not necessarily represent those of their affiliated organizations, or those of the publisher, the editors and the reviewers. Any product that may be evaluated in this article, or claim that may be made by its manufacturer, is not guaranteed or endorsed by the publisher.

## Supplementary Material

Supplementary Figure 1 Kaplan–Meier survival curves for OS according to **(A)** education level, **(B)** income, **(C)** unemployment rate, and **(D)** poverty rate of the patients. OS, overall survival; U/M, uninsured/Medicaid; NHW, non-Hispanic White; NHB, non-Hispanic Black; NHAPI, non-Hispanic Asian or Pacific Islander.

Supplementary Figure 2 Kaplan–Meier survival analysis for OS in subgroups as follows: **(A)** age > 65 years, **(B)** non-Hispanic White, **(C)** unmarried status, **(D)** uninsured or Medicaid status, **(E)** SEER distant stage, **(F)** AJCC stage IV, **(G)** Grade II–IV tumor, and **(H)** metastatic status, stratified by education level. OS, overall survival; AJCC, American Joint Committee on Cancer; Education level: Q1 (low level, lowest quartile), Q2 (moderate level, 2nd and 3rd quartiles), and Q3 (high level, highest quartile).

Supplementary Figure 3 Kaplan–Meier survival analysis for CSS in subgroups as follows: **(A)** age > 65 years, **(B)** non-Hispanic White, **(C)** unmarried status, **(D)** uninsured or Medicaid status, **(E)** SEER distant stage, **(F)** AJCC stage IV, **(G)** Grade II–IV tumor, and **(H)** metastatic status, stratified by education level. CSS, cancer-specific survival; AJCC, American Joint Committee on Cancer; Education level: Q1 (low level, lowest quartile), Q2 (moderate level, 2nd and 3rd quartiles), and Q3 (high level, highest quartile).

Supplementary Table 1 Univariate and multivariate logistic regression analysis of radiotherapy receipt in AJCC stage IV patients. OR, odd ratio; CI, confidence interval; NHW, non-Hispanic White; NHB, non-Hispanic Black; NHAPI, non-Hispanic Asian or Pacific Islander.
