# Supplementary figures and images for: Associations of Education Level With Survival Outcomes and Treatment Receipt in Patients With Gastric Adenocarcinoma
Source: Front Public Health. 2022 Jun 9;10:868416. doi: 10.3389/fpubh.2022.868416 (PMC9218109; doi:10.3389/fpubh.2022.868416)

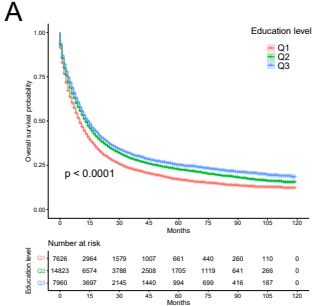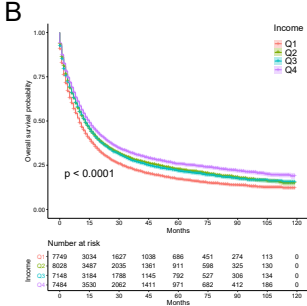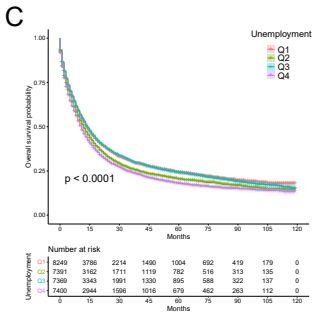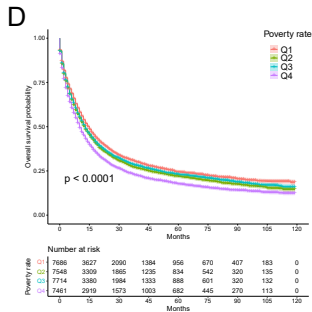

Supplement: Supplementary Figure 1 — Kaplan–Meier survival curves for OS according to (A) education level, (B) income, (C) unemployment rate, and (D) poverty rate of the patients. OS, overall survival; U/M, uninsured/Medicaid; NHW, non-Hispanic White; NHB, non-Hispanic Black; NHAPI, non-Hispanic Asian or Pacific Islander. [file Image_1.PDF]

A

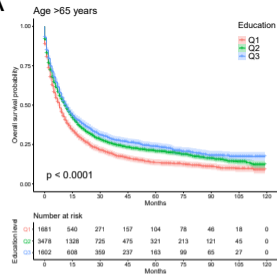

B

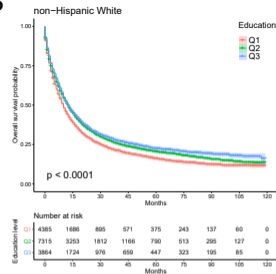

C

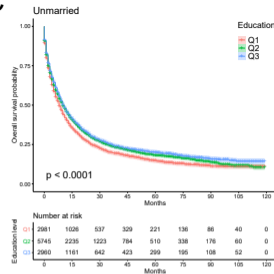

D

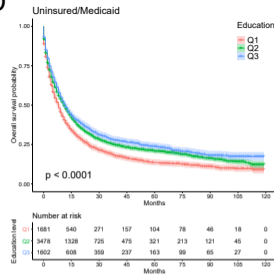

E

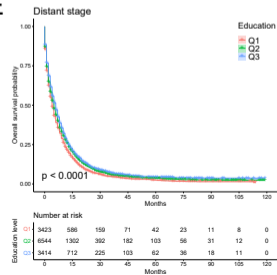

F

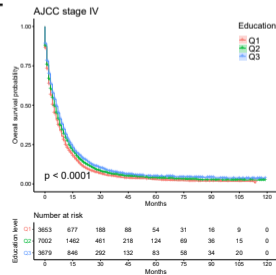

G

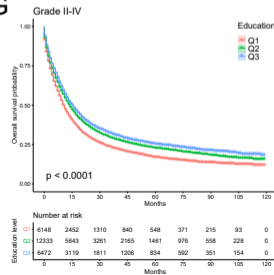

H

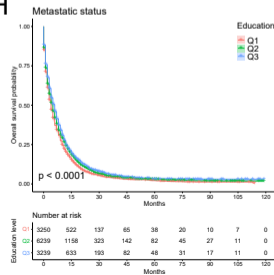

Supplement: Supplementary Figure 2 — Kaplan–Meier survival analysis for OS in subgroups as follows: (A) age > 65 years, (B) non-Hispanic White, (C) unmarried status, (D) uninsured or Medicaid status, (E) SEER distant stage, (F) AJCC stage IV, (G) Grade II–IV tumor, and (H) metastatic status, stratified by education level. OS, overall survival; AJCC, American Joint Committee on Cancer; Education level: Q1 (low level, lowest quartile), Q2 (moderate level, 2nd and 3rd quartiles), and Q3 (high level, highest quartile). [file Image_2.PDF]

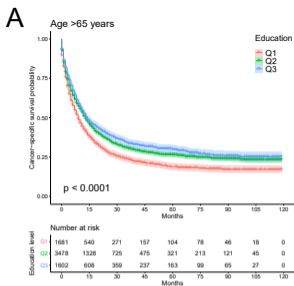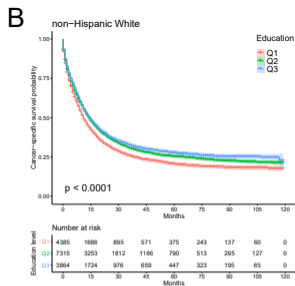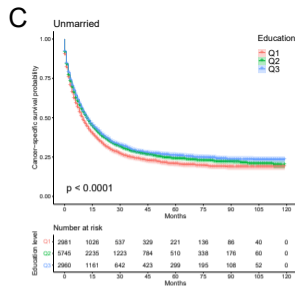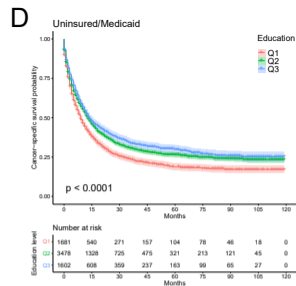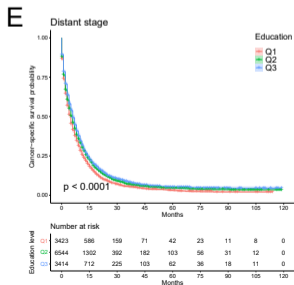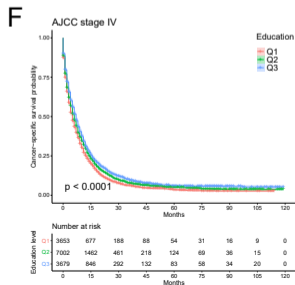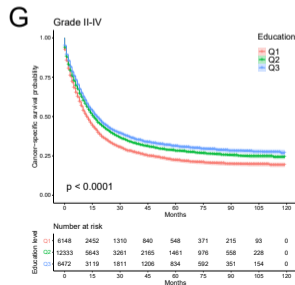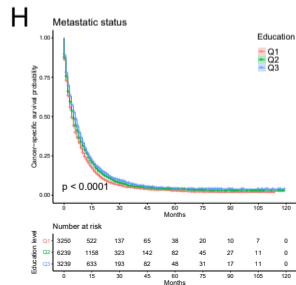

Supplement: Supplementary Figure 3 — Kaplan–Meier survival analysis for CSS in subgroups as follows: (A) age > 65 years, (B) non-Hispanic White, (C) unmarried status, (D) uninsured or Medicaid status, (E) SEER distant stage, (F) AJCC stage IV, (G) Grade II–IV tumor, and (H) metastatic status, stratified by education level. CSS, cancer-specific survival; AJCC, American Joint Committee on Cancer; Education level: Q1 (low level, lowest quartile), Q2 (moderate level, 2nd and 3rd quartiles), and Q3 (high level, highest quartile). [file Image_3.PDF]
