# Supplementary material for: Associations of Education Level With Survival Outcomes and Treatment Receipt in Patients With Gastric Adenocarcinoma
Source: Front Public Health. 2022 Jun 9;10:868416. doi: 10.3389/fpubh.2022.868416 (PMC9218109; doi:10.3389/fpubh.2022.868416)
Supplement: Supplementary Table 1 — Univariate and multivariate logistic regression analysis of radiotherapy receipt in AJCC stage IV patients. OR, odd ratio; CI, confidence interval; NHW, non-Hispanic White; NHB, non-Hispanic Black; NHAPI, non-Hispanic Asian or Pacific Islander. [file Table_1.PDF]

Table S1 Univariate and multivariate logistic regression analysis of radiotherapy receipt in AJCC stage IV patients.

Characteristics	Levels	Crude OR	95% CI	*P* value	Adjusted OR	95% CI	*P* value
**Age**	≤65 years	Ref			Ref		
	>65 years	0.784	0.720-0.854	<0.001	0.702	0.639-0.772	<0.001
**Sex**	Male	Ref			Ref		
	Female	0.655	0.597-0.718	<0.001	0.701	0.636-0.774	<0.001
**Race**	NHW	Ref			Ref		
	Hispanic	0.617	0.551-0.690	<0.001	0.715	0.631-0.810	<0.001
	NHAPI	0.839	0.739-0.952	0.007	0.925	0.804-1.064	0.274
	NHB	0.714	0.624-0.817	<0.001	0.738	0.638-0.853	<0.001
**Insurance**	Insured	Ref			Ref		
	Medicaid	0.799	0.714-0.894	<0.001	0.915	0.810-1.035	0.158
	Uninsured	0.652	0.535-0.794	<0.001	0.653	0.530-0.804	<0.001
**Marriage**	Married	Ref			Ref		
	Divorced	0.974	0.840-1.130	0.728	0.995	0.853-1.160	0.946
	Single	0.771	0.686-0.868	<0.001	0.849	0.749-0.963	0.011
	Widowed	0.614	0.530-0.710	<0.001	0.772	0.657-0.905	0.001
**SEER stage**	Distant	Ref			Ref		
	Regional	2.883	2.560-3.246	<0.001	0.859	0.640-1.154	0.312
**Tumor grade**	I	Ref					
	II	0.863	0.628-1.187	0.365	0.846	0.608-1.177	0.320
	III	0.693	0.509-0.944	0.020	0.658	0.478-0.908	0.011
	IV	0.944	0.604-1.475	0.800	0.802	0.504-1.278	0.354
	Unknown	0.577	0.418-0.795	0.001	0.612	0.439-0.854	0.004
**Metastasis**	No	Ref			Ref		
	Yes	0.324	0.290-0.362	<0.001	0.261	0.198-0.344	<0.001
**Income**	Q1 (lowest)	Ref			Ref		
	Q2	0.692	0.614-0.779	<0.001	0.793	0.670-0.940	0.007
	Q3	1.003	0.893-1.126	0.963	0.737	0.599-0.906	0.004
	Q4 (highest)	0.871	0.774-0.980	0.021	0.494	0.378-0.645	<0.001
**Unemployment**	Q1 (lowest)	Ref			Ref		
	Q2	0.962	0.859-1.077	0.503	1.001	0.878-1.142	0.988
	Q3	0.606	0.536-0.685	<0.001	0.754	0.639-0.890	0.001
	Q4 (highest)	0.879	0.784-0.985	0.027	0.988	0.835-1.169	0.888
**Poverty rate**	Q1 (lowest)	Ref			Ref		
	Q2	0.987	0.880-1.107	0.823	0.882	0.772-1.009	0.067
	Q3	0.663	0.587-0.749	<0.001	0.618	0.496-0.771	<0.001
	Q4 (highest)	0.909	0.809-1.022	0.110	0.640	0.506-0.808	<0.001
**Education level**	Q1 (low)	Ref			Ref		
	Q2 (moderate)	0.878	0.793-0.972	0.012	1.034	0.892-1.198	0.658
	Q3 (high)	0.967	0.862-1.086	0.574	1.209	0.990-1.476	0.063

Abbreviations: OR, odd ratio; CI, confidence interval; NHW, non-Hispanic White; NHB, non-Hispanic Black; NHAPI, non-Hispanic Asian or Pacific Islander.
